# Modern Sociology

**Published:** 1902-05-10

**Authors:** 


### MODERN SOCIOLOGY.

### SPECIAL SCHOOLS UNDER THE LONDON SCHOOL BOARD.

### IV.—FOR THE BLIND.

ALTHOUGH blind children were the first of the defective classes to be dealt with by the School Board for London in special schools, it is a curious fact that at the present time the schools for the blind are behind the others in method and progress. Reference has already been made to the system in force, namely, that blind children are being taught by blind teachers, who have only young sighted monitors to assist them. This does not appear to be at all a satisfactory condition of things, though we would not for a moment be supposed to detract from the excellence of the individual teachers, some of whom are first rate as far as, with such a defect, it is possible for them to be. It is the system that appears to us to be radically wrong, and it is to be hoped that the recent appointment of four sighted teachers is the beginning of a better state of things.

It was as early as 1874 that the School Board attempted to deal with the education of blind children, who up till that time had been left entirely in the hands of voluntary agencies. It is true that the law of compulsory education applied no less to the blind than to the sighted; but this was simply because no special provision was made by it for defective children; and it would have been useless to compel these children to attend school when there was nothing for them but the ordinary routine of school work. Notwithstanding this obvious fact, however, the Board of 1874 determined to ask the divisional committees to take steps to induce the blind children to attend the ordinary schools, and resolved, further, that the "Society for Providing Home Instruction for the Blind" should be informed of the fact, so that it might provide special instruction for these children. The results, however, appear to have been unsatisfactory, and next year the Board appointed peripatetic teachers, to instruct the blind in the ordinary schools. At the end of four years the Education Department was persuaded to recognise what was being done; and the Board at the same time agreed to recognise the attendances of these children. Centres were then organised and supplied with the necessary books and apparatus.

From 1885 to 1889 a Royal Commission sat to inquire, among other things, into the education of the blind and deaf as practised in England. At the end of its inquiries it recommended that the education of the blind should be placed under State control, instead of being left in the hands of voluntary agencies or the Poor Law Guardians; and, further, that the age up to which such children were to be dealt with should be raised to sixteen.

The next step was taken four years later, twenty years after the State's first attempt to deal with the problem, and when two generations of blind scholars had lent themselves for experiment to the powers that be. In 1893 the duty was imposed upon the school authorities—in the case of London this was the School Board—of providing elementary education, including industrial training, to the blind and deaf, in some school certified as suitable by the Education Department. Practically, it may be said that what the voluntary agencies had recognised all along, namely, the necessity of training the blind to earn a living, came at last to be recognised as a duty of the State. For, although we owe it to voluntary agencies that blind readers of the Braille scriptures still sit under archways and at street corners, monuments to their own incapacity to earn a living by any better means than thinly disguised begging, yet it is a fact that for many years this necessity has been recognised, by more than one school. The School for the Indigent Blind exists "to render the blind self-reliant by teaching them a trade." It has an interesting history. At the close of the eighteenth century, seven gentlemen visited Paris, and were much impressed by the work among the blind by Valentine Haüy; on returning home they inspired the nation with the necessity of establishing training schools for the blind. Large sums were contributed, and the School for the Indigent Blind was founded, on the site of the Dog and Duck tea-gardens, St. George's Fields, in 1799. The charity was granted a Royal Charter in 1826, but it remained, and remains to this day, a charity. This is not the place to describe the splendid work being done under the direction of the Principal, the Rev. St. Clare Hill, who has lifted the school on to a high level of efficiency; but it may be noted that in 1893, on the passing of the Act referred to above, this school abandoned its work for the young blind, and now devotes itself to the training of boys and girls over twelve, and to finding employment for them as soon as they are capable of carrying on a trade. The school, which is on the point of moving to palatial new premises at Leatherhead, has taught more than 3,500 blind in its century of work, and there are now 200 pupils, who, for a period of six years, are being educated, clothed, maintained, and taught a trade. All the teachers are sighted. Another somewhat similar school is that at 10 Upper Avenue Road, Hampstead. It has a cumbersome name—"London Society for Teaching the Blind to Read, and Training them in Industrial Occupations." Here, as elsewhere, a great point is made of music. Here, again, all the teachers are sighted (with the exception of one, who is an old pupil).

If the classes for special instruction under the Board were in any efficient sense preparatory to the course of training at such schools as these, an assured future would open up before those blind children who have the brains to learn a trade. As long, however, as two systems at variance with each other are in force, this does not appear to be possible.

The course of instruction under the Board consists of reading by the Braille method, writing in Braille characters, the very cumbersome system of arithmetic which appears to be the only one known to the blind, besides weaving, knitting, typewriting, and, latterly, for elder girls, cookery and laundry work. The latter we have not seen, but the former was a painful exhibition of lack of power combined with the "willingness of the spirit."

There appear to be more openings for boys than for girls among the blind. For the former, basket and mat weaving, in all their various and highly-skilled branches, absorb the larger number of the adult blind; piano tuning, organ playing, and musical careers generally are possible; and there seems to be no reason whatever why women should not be trained as piano tuners as well as men. Indeed, on mentioning this to the head of a well-known institution for the blind, we were assured that it was only the certainty of demand that was wanted. As things are, blind women can earn a poor living by knitting, light weaving (real mat and basket making is very heavy work), but the number of trades they are at present taught seems to be singularly limited.

It is worth while to notice that in Japan, massage was until recently entirely in the hands of blind people; and of 1,000 persons thus engaged in Yokohama to-day only 100 are sighted.

